# A literature review of first-episode psychosis, a perspective on the future

**DOI:** 10.1192/j.eurpsy.2025.2050

**Published:** 2025-08-26

**Authors:** M. C. Herrero Rodriguez, Z. Martin García, C. Vall García, J. M. Martínez Sánchez

**Affiliations:** 1 Psychiatry, Río Hortega University Hospital, Valladolid, Spain

## Abstract

**Introduction:**

First-episode psychosis is one of the major challenges of mental health research worldwide because it is a traumatic experience for patients and their families. Patients who experience these episodes may experience fear, distress, and isolation.

**Objectives:**

The early phase of psychosis is a critical period when long-term outcome is predictable and biological, psychological and psychosocial influences are developing and display maximal plasticity. This phase presents important opportunities for secondary prevention and delaying treatment may affect the chance of recovery. The main goal is to reduce the duration of untreated psychosis and ensure that, in addition to symptom remission, there is also psychosocial recovery. Currently, the clinical and research focus in psychotic disorders has shifted toward first episode psychosis, early detection of the prodromal phase of psychosis, and an effective integrated treatment model known as “Early Intervention.”

**Methods:**

Selective review of the literature on first episode psychosis.

**Results:**

The studies strongly support the efficacy of antipsychotic medication as both acute and maintenance treatment for patients with a first episode of psychosis.

**Conclusions:**

Early intervention may improve outcomes in first episode psychosis. The use of new antipsychotics with greater efficacy and fewer side effects may improve medication adherence and reduce morbidity associated with repeated relapses. However, the optimal duration of maintenance treatment has not been determined and a long duration of untreated psychosis may be associated with a poorer treatment response. Finally, services for Early Intervention should be easily accessible, non-threatening and non-stigmatising.

**Disclosure of Interest:**

None Declared

